# The Neurogenic Factor *NeuroD1* Is Expressed in Post-Mitotic Cells during Juvenile and Adult Xenopus Neurogenesis and Not in Progenitor or Radial Glial Cells

**DOI:** 10.1371/journal.pone.0066487

**Published:** 2013-06-14

**Authors:** Laure Anne D'Amico, Daniel Boujard, Pascal Coumailleau

**Affiliations:** 1 Centre de Ressources Biologiques Xénope UMS U3387, CNRS, and Rennes 1 University, Rennes, France; 2 IRSET U1085, INSERM and Rennes1 University, Rennes, France; University of Colorado, Boulder, United States of America

## Abstract

In contrast to mammals that have limited proliferation and neurogenesis capacities, the *Xenopus* frog exhibit a great potential regarding proliferation and production of new cells in the adult brain. This ability makes *Xenopus* a useful model for understanding the molecular programs required for adult neurogenesis. Transcriptional factors that control adult neurogenesis in vertebrate species undergoing widespread neurogenesis are unknown. *NeuroD1* is a member of the family of proneural genes, which function during embryonic neurogenesis as a potent neuronal differentiation factor. Here, we study in detail the expression of *NeuroD1* gene in the juvenile and adult *Xenopus* brains by in situ hybridization combined with immunodetections for proliferation markers (PCNA, BrdU) or in situ hybridizations for cell type markers (*Vimentin, Sox2*). We found *NeuroD1* gene activity in many brain regions, including olfactory bulbs, pallial regions of cerebral hemispheres, preoptic area, habenula, hypothalamus, cerebellum and medulla oblongata. We also demonstrated by double staining *NeuroD1*/BrdU experiments, after long post-BrdU administration survival times, that *NeuroD1* gene activity was turned on in new born neurons during post-metamorphic neurogenesis. Importantly, we provided evidence that *NeuroD1*-expressing cells at this brain developmental stage were post-mitotic (PCNA-) cells and not radial glial (*Vimentin*+) or progenitors (*Sox2*+) cells.

## Introduction

Adult neurogenesis is a fascinating biological trait, which has captivated researchers since many years. In mammals and under normal conditions, adult neurogenesis has been identified in two anatomical regions: the subventricular zone (SVZ) lining the lateral ventricles and the subgranular zone (SGZ) of the hippocampal dentate gyrus (reviewed by [1]). Interestingly, adult neurogenesis seems to be more abundant in birds, reptiles, amphibians and fish than in mammals (reviewed by [2]–[5]. Recently, the detailed neuroanatomical mappings of proliferative activity in the adult brain were provided in two non-mammalian vertebrate models, the fish *Danio rerio* (reviewed in [6]–[8] and the amphibian *Xenopus laevis* [9]. In these two vertebrates, a widespread proliferation and neurogenic activities were detected. In the adult fish *Danio rerio* brain, more than ten distinct adult proliferative zones have been identified along the whole brain axis in areas such as olfactory bulb, telencephalon, thalamus, epithalamus, preoptic region, hypothalamus, tectum, cerebellum, rhombencephalon, and spinal cord [10], [11]. In the zebrafish, several studies have demonstrated that radial progenitors exhibit proliferative activity and give birth to at least part of the newborn neurons [12]. In post-metamorphic *Xenopus laevis* brain, at both juvenile and adult stages, cell proliferation activity was also found in various brain regions, namely, olfactory bulbs, cerebral hemispheres, preoptic region, ventral hypothalamus and cerebellum [9]. Most importantly, new differientiated neurons and oligodendrocytes were clearly detected at these stages [9]. These studies fully confirmed that amphibian and fish adult brains are characterized by a greater number of proliferation and neurogenic compartments than previously described in other vertebrates. However, the molecular mechanisms, and among them the transcriptional program, underlying the widespread neurogenesis found in adult frog and fish brains remains unknown.

Studies in mammals have indicated that several transcription factors of the basic helix-loop-helix (bHLH) family play critical roles not only during during embryonic neurogenesis but also during post-natal neurogenesis [13], [14]. In an effort to identify transcription factors involved in *Xenopus* post-metamorphic neurogenesis, we have focused our analysis on the NeuroD1 factor, also known as NeuroD or Beta2. NeuroD1 is a proneural basic helix-loop-helix (bHLH) transcription factor that belongs to the Ath (*Drosophila Atonal*) group, which also includes Math and Neurogenin subfamilies (reviews in [15], [16]. In *Xenopus* embryo, all primary neurons, neurogenic placodes, and retina strongly express the neuronal diffentiation gene *NeuroD1* [17], [18]. When ectopically expressed in *Xenopus* embryos, NeuroD1 can convert non-neuronal ectodermal cells into fully differentiated neurons, indicating that it can be a potent neuronal differentiation factor [17]. In mammals, *NeuroD1* is widely expressed during brain development and is essential for the development of the central nervous sytem, particularly for the generation of granule cells in the hippocampus and cerebellum [19], [20].

To gain further insight into the transcriptional program that controls adult neurogenesis in non-mammalian vertebrates, this study examined in details the *NeuroD1* gene expression, together with various cellular markers (*Vimentin*, *Sox2*, PCNA and BrdU), in post-metamorphic (juvenile and adult) *Xenopus* brains. The data show that a high number of *NeuroD1*-expressing cells can be detected in various brain areas and that *NeuroD1* gene is up-regulated during post-metamorphic neurogenesis. Moreover, we provide evidence that *NeuroD1* is expressed in post-mitotic (PCNA-) neuronal cells and not in radial glial (*Vimentin*+) or neural progenitors (*Sox2*+) cells.

## Results

### Strong and spatially restricted expression of the *NeuroD1* gene in juvenile and adult *Xenopus* brains

**Figure 1:**
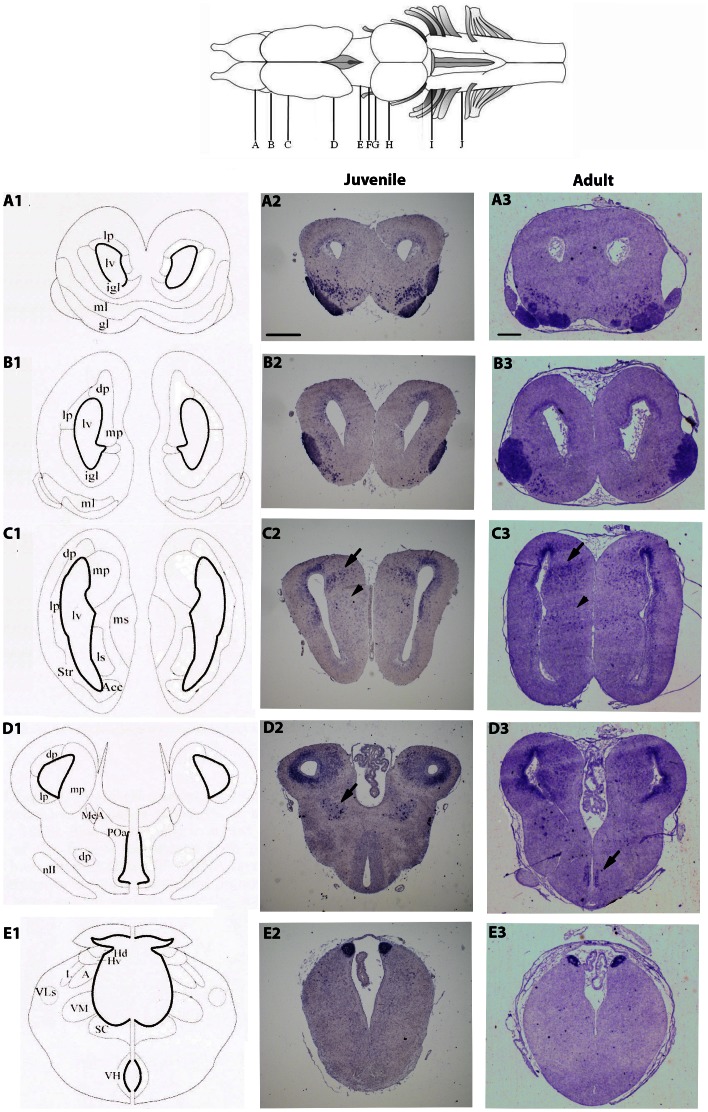
Expression pattern of *NeuroD1* in the juvenile (A2–E2) and adult (A3–E3) *X. laevis* brains. A1–E1) Schematic coronal illustrations of the corresponding transverse sections of a juvenile *X. laevis* brain (NF stage 66). The drawing at the top of the figure shows a dorsal view of the *X. laevis* brain. The letters correspond to the rostro-caudal location of sections as depicted in the whole brain drawing. Arrows and arrowheads in C2, C3, D2, and D3 highlight less conspicuous areas of labeling. Abbreviations are defined in Table 1. The anatomical drawings are from [55], with modifications of basal ganglia subdivisions according to [56]. For all images, dorsal is to the top. Scale bar = 400 µm in A2–E2, and 100 µm in A3–E3.

**Figure 2:**
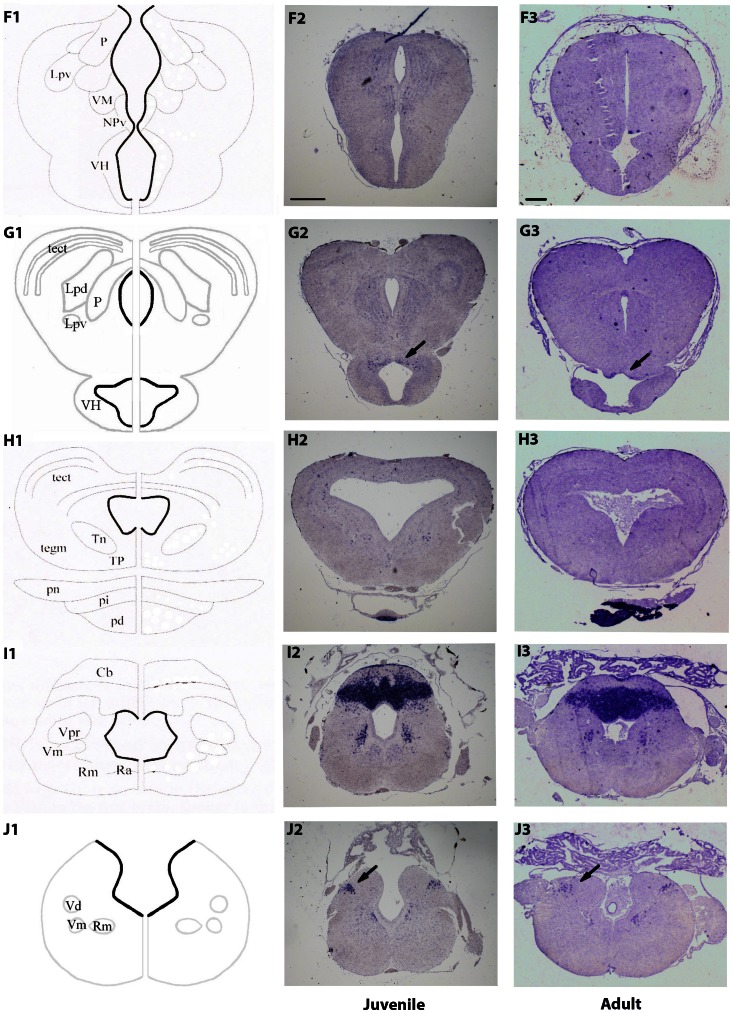
Expression pattern of *NeuroD1* in the juvenile (F2–J2) and adult (F3–J3) *X. laevis* brains. (F1–J1) Schematic coronal illustrations of the corresponding transverse sections of a juvenile *X. laevis* brain (NF stage 66). The letters correspond to the rostro-caudal location of sections as depicted in the whole brain drawing (see Figure 1). Arrows and arrowheads in G2, G3, J2 and J3 highlight less conspicuous areas of labeling. Abbreviations are defined in Table 1. The anatomical drawings, with the exceptions of G1 and J1, are from [55], with modifications of basal ganglia subdivisions according to [56]. For all images, dorsal is to the top. Scale bar = 400 µm in F2–J2, and 100 µm in F3–J3.

In order to identify the brain sub-divisions that express the *NeuroD1* gene, in situ hybridization experiments, using high stringent conditions, were performed on coronal sections throughout the whole juvenile and adult brains. The detailed *NeuroD1* expression pattern is illustrated in figures 1 and 2 and described below. There were no significant differences between males and females in any brain regions.

**Table 1:** Abbreviations of major neuro-anatomical landmarks.

A	anterior thalamic nucleus	P	posterior thalamic nucleus
Acc	nucleus accumbens	pd	pars distalis
Cb	cerebellum	pi	pars intermedia
dp	dorsal pallium	pn	pars nervosa
gl	glomerular layer	POA	preoptic area
Hd	dorsal habenular nucleus	Ra	raphe nucleus
Hv	ventral habenular nucleus	Rm	nucleus reticularis medius
igl	internal granule cell layer	SC	suprachiasmatic nucleus
L	lateral thalamic nucleus	Str	striatum
lp	lateral pallium	tect	optic tectum
Lpv	lateral thalamic nucleus	tegm	mesencephalic tectum
ls	lateral septum	Tn	tegmental nuclei
lv	lateral ventricle	TP	posterior tuberculum
MeA	medial amygdala	VH	ventral hypothalamic nucleus
ml	mitral layer	VLs	superficial ventral nucleus
mp	medial pallium	VM	ventromedial thalamic nucleus
ms	medial septum	Vm	nucleus motorius nervi trigemini
nII	cranial nerve II	Vpr	nucleus sensorius principalis nervi trigemini

In the more rostral portions of the telencephalon, *NeuroD1* expression was strongly detected in the glomerular and mitral cell layers of the olfactory bulbs (Figs. 1, A1–A3 and B1–B3). No expression of *NeuroD1* was detected in the inner granule cell layer. Moving caudally towards mid-telencephalic levels, *NeuroD1*-positive cells were predominantly found in the dorsal, lateral and medial (arrows) territories of the pallia (Figs. 1, B1–B3 and C1–C3). Scattered cells could be also observed in the medial septum (arrowheads in Fig. 1, C2 and C3). No *NeuroD1* labeling was observed in ventricular cells directly at the ventricle but rather in the migrated cells in the mantle zone. In more caudal portions of the telencephalon, *NeuroD1*-expressing cells were still detected in the dorsal, lateral and medial pallium (Figs. 1, D1–D3). In addition, *NeuroD1* was expressed in part of the medial amygdala and bed nucleus striae medullaris (arrow in Figs. 1, D2). Ventrally, *NeuroD1*-expressing cells were also present in the medial portion of the anterior preoptic telencephalic area, such labeling being manifest in the adult brain section (arrow in Figs. 1, D3). In both pallial and preoptic areas, numerous *NeuroD1*-expressing cells were localized close to the ventricles, mainly in the subventricular layers from where the newborn post-mitotic neuronal cells migrate to their more peripheral final destination.

In the diencephalon, the level of *NeuroD1* expression was moderate compared to telencephalon. The most densely labeled cell group was located in the epithalamus, namely, in the dorsal and ventral nuclei of the habenula (Figs. 1, E1 and E2). This expression pattern was maintained at adult stage (Figs. 1, E3). More caudally, a low number of moderately labeled cells was also found in posterior and ventromedial thalamic areas (Fig. 2, F2), which was no longer detected at adult stage (Fig. 2, F3). In addition, a discrete cell population located dorsally in the hypothalamic region, and very close to the infundibular recess, obviously expressed *NeuroD1* in both juvenile and adult brains (arrows in Figs. 2, G1–G3). At mesencephalic level, only very few and dispersed *NeuroD1*-expressing cells were present in the tectum and tegmentum at juvenile stage (Figs. 2, H2). This weak labeling was not found in corresponding adult mesencephalic sections (Fig. 2, H3).

Within the metencephalon, very high levels of *NeuroD1* labeling were found in the region of the cerebellum, such expression being restricted to the granular layer (Figs. 2, I1–I3). Interestingly, this strong *NeuroD1* expression was maintained in adult cerebellum (Figs. 2, I3). Few *NeuroD1*-expressing cells were also found in areas corresponding to the nuclei of the trigeminal nerves (Figs. 2, I1 and I3). Posterior to the cerebellum, in the medulla oblongata (Figs. 2, J1–J3), *NeuroD1* expression was still detected in nuclei of trigeminal nerves and, more dorsally, close to the lateral vestibular area (arows in Figs. 2, J2 and J3). Importantly, throughout the juvenile and adult brains, we never observed *NeuroD1*-expressing cells in the ependymal layer.

### *NeuroD1*-expressing cells are not radial glial or progenitors cells

**Figure 3:**
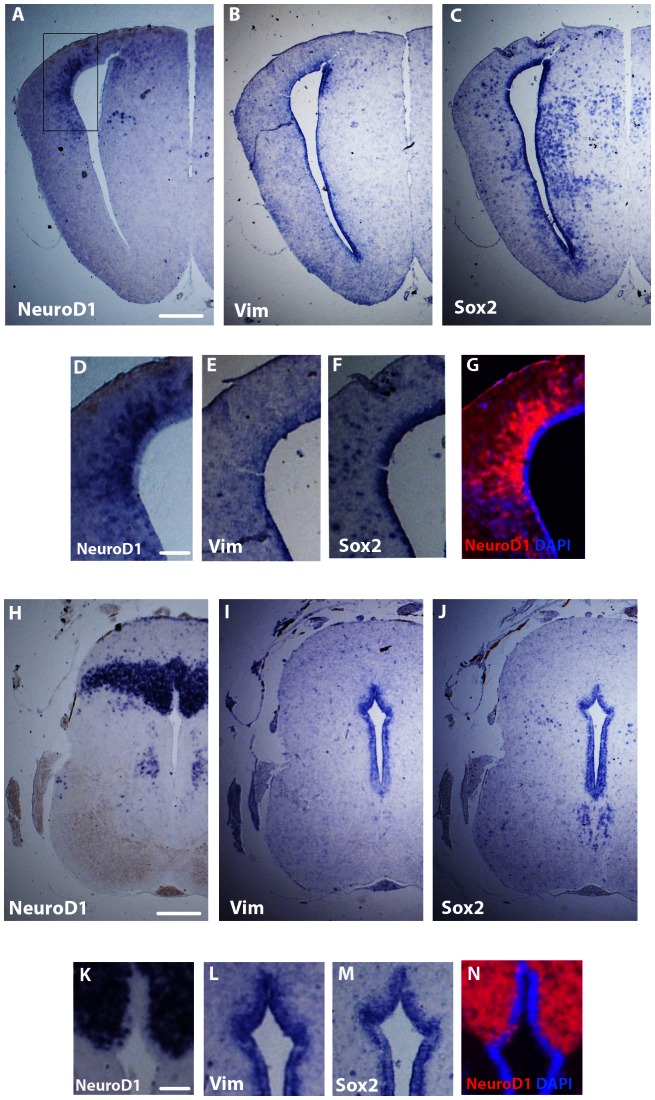
Expression patterns of *NeuroD1*, *Sox2* and *Vimentin* in the juvenile *X. laevis* brain. In situ hybridizations on coronal sections of cerebral hemispheres (A–G) and cerebellum (H–N). In cerebral illustrations, D, E and F are high magnifications of A, B and C, respectively. In cerebellum illustrations, K, L and M are high magnifications of H, I and J, respectively. To allow merge with the DAPI staining, colors of high magnification illustrations D and K were negatively inverted in photos G and N, respectively. For all images, dorsal is to the top. Scale bar = 220 µm in A–C and H–J; 95 µm in D–G and K–N.

As previously described, *NeuroD1*-positive cells could be observed both in the parenchyma and in the subventricular layers, but never in the ventricular zone adjacent to the ventricle. To more precisely define the cellular expression of *NeuroD1* within the brain, serial in situ hybridizations were performed on thin adjacent sections using the *NeuroD1* probe, but also *Vimentin* and *Sox2* probes, as markers of radial glial cells and neural progenitor cells, respectively [21], [22]. Radial glial cells, known to behave as neural stem cell (reviewed in Kriegstein and Alvarez-Buylla, 2009), were previously identified in the ventricular layers of post-metamorphic *Xenopus* brain [9]. We focused our analyses on the pallial and cerebellar regions of juvenile brains because these two regions displayed heavy *NeuroD1* expressions (see Figs. 1 and 2). As confirmed in figure 3, strong *NeuroD1* expression domains were detected in the dorso-lateral pallium (Fig. 3, A) and in the cerebellum (Fig. 3, H). Using adjacent sections, in situ hybridizations with *Vimentin* (Fig. 3, B and I) and *Sox2* (Fig. 3, C and J) probes showed strong expressions of both genes in ventricular cells of the pallium and sub-pallium. *Sox2* labeling was also detected in few cells localized in the parenchyma (Fig. 3, C and J). Most importantly, higher magnifications of the dorso-lateral pallium (Figs. 3, D–F) and the granular layer of the cerebellum (Figs. 3, K–M) provided evidence that *NeuroD1*-positive cells were detected in large amount outside the ventricular layer cells that expressed *Vimentin* and *Sox2* markers (compared Figs. 3 D to E and F; Figs. 3 K to L and M). These observations were reinforced by performing double labelings *NeuroD1*/DAPI on the same sections (Figs. 3, G and N). Taken together, these in situ hybridization experiments demonstrated that expression domains of *NeuroD1* were excluded from the ventricular layers as these domains did not overlap with radial glia or neural progenitor markers.

### *NeuroD1*-expressing cells are post-mitotic

**Figure 4:**
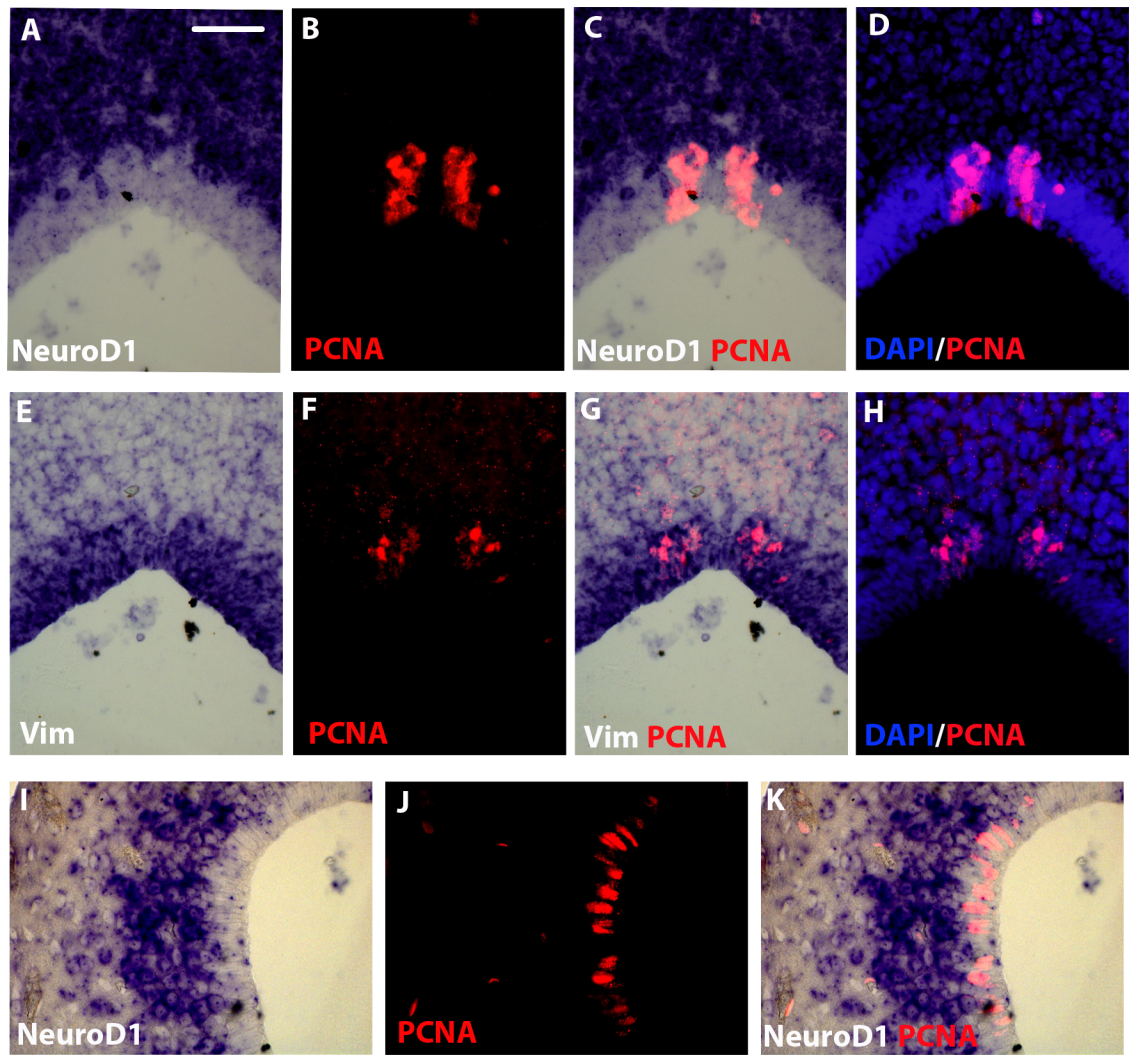
*NeuroD1*/PCNA and *Vimentin*/PCNA double stainings in the cerebellar and pallial regions of juvenile *X. laevis* brain. Coronal sections at the level of cerebellum (A–H) or pallium (I–K). In situ hybridization using a *NeuroD1* (A–D and I–K) or a *Vimentin* (E–H) probe combined with PCNA immunohistochemistry. For all images, dorsal is to the top. Scale bar = 30 µm.

Our detailed analysis of *NeuroD1* gene activity have revealed that, throughout juvenile and adult brains, *NeuroD1* transcripts were never detected in the ventricular layers, where both neural stem cells and mitotic neuronal precursor cells were located (Figure 3). This consistent *NeuroD1* expression pattern, together with our previous published data that identified proliferating cells specifically in the ventricular layers of juvenile and adult brains [9], strongly suggested that *NeuroD1*-expressing cells were post-mitotic cells. To further investigate this hypothesis, we designed double labeling experiments combining *NeuroD1* RNA in situ hybridization and immunocytochemistry for the PCNA proliferation marker on coronal sections of pallial and cerebellar regions (Figure 4). As shown at high magnifications of the cerebellum (Fig. 4, A–H), the PCNA positive cells were clearly found restricted to the ventricular layer (Fig. 4, B–D and F–H) while *NeuroD1*-expressing cells were observed both in sub-ventricular layers and parenchyma (Fig. 4, A and C). No significant co-localization of *NeuroD1* transcripts and PCNA in the same cell could be detected (Fig. 4C). As expected, using adjacent coronal section, PCNA-positive cells perfectly co-localized with *Vimentin*-expressing cells in the ventricular layer (Figs. 4, E–H). Using tissue sections of the pallial region, we also revealed *NeuroD1* expression in post-mitotic cells, i.e outside the ventricular zone containing PCNA-positive cells (Fig. 4, I–K). Identical results were obtained using the BrdU proliferation marker after short (2 days) post-BrdU administration survival time (data not shown). Overall, our data demonstrated that *NeuroD1* expression in juvenile *Xenopus* brain was only detected in post-mitotic cells undergoing neuronal differentiation, supporting the previous observation that *NeuroD1* expression was not detected in progenitor and radial glial cells.

### *NeuroD1* gene is up-regulated in new born cells during post-metamorphic neurogenesis

**Figure 5:**
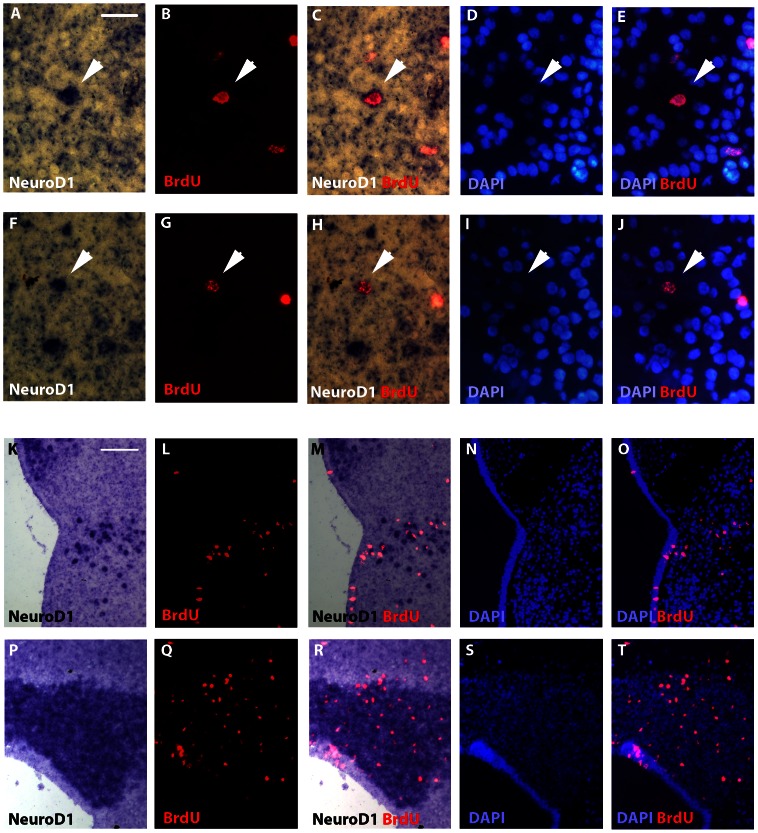
*NeuroD1*/BrdU double stainings on telencephalic and cerebellar sections of a juvenile *X. laevis* brain. (A–J) Telencephalon high magnifications of *NeuroD1* in situ hybridizations (A, C, F and H) combined with BrdU immunodetections (B, C, F and H) after 14-days BrdU post-administration time. Arrows indicate double stained cells. DAPI stainings are indicated to certify the presence of the nucleus. (K–T) Low magnifications of the above *NeuroD1*/BrdU/DAPI triple labelling experiments showing larger view of telencephalon (K–O) and cerebellum (P–T). For all images, dorsal is to the top. Scale bar = 15 µm in A–J; 45 µm in K–T.

We additionally asked wether *NeuroD1* transcription was turned on in post-metamorphic neurogenesis, i.e during the differentiation process of juvenile new born cells. In order to adress this question, we followed the fate of proliferative cells 14 days after injections of BrdU at juvenile stage. In situ hybridizations experiments for *NeuroD1* transcripts combined with immunodectection for BrdU were then performed on thin telencephalic and cerebellar coronal sections. In the telencephalon, 14 days post-injections, BrdU-positive cells were found in large amount outside the ventricular layers in the medial and lateral septum, striatum and nucleus accumbens, and to a lesser extent in the pallium (Fig. 5, K–O and D'Amico et al., 2011). In the cerebellum, BrdU-positive cells were mainly detected in the granular layer (about 90%) and very few were found in the molecular layer (Fig. 5, P–T; D'Amico et al., 2011). In addition, both in telencephalon and cerebellum, the majority of BrdU-positive cells were also observed in regions with high cell density (see Fig. 5, O and T). In order to make undoubtedly *NeuroD1*/BrdU cellular co-localization analysis, we focused our double staining illustrations on the medial pallium area because *NeuroD1* expressing cells were found more scattered than in the dorso-lateral pallium and cell density was also weak. As shown by the high magnification images of the figure 5 (A–J), we found individual BrdU- positive cells (Fig. 5, B and G) that perfectly matched with nuclei stained with DAPI (Fig. 5, D–E and I–J). Most importantly, a strong *NeuroD1* expression was detected in some of these BrdU-positive cells (arrowheads in Fig. 5, A–C and F–H). Cell counting analysis in these low cell density areas revealed that approximately 1.2% of the cells were labeled for both *NeuroD1* and BrdU. Unfortunately, the experimental procedure did not allow to quantify double-positive cells in high cell density areas (Fig. 5, N and S), in particular in the granular layer of cerebellum where strong *NeuroD1* gene activity and BrdU labelling were detected (Figure 5, P–T). Nevertheless, these data demonstrated that new born cells in the medial pallium of a juvenile brain can turn on *NeuroD1* gene expression during the neuronal differentiation process.

## Discussion

To gain further insight into the activation of *NeuroD1* gene in non mammalian vertebrates, we examined the precise expression pattern of *NeuroD1* by in situ hybridization on coronal sections throughout the whole juvenile and adult *Xenopus laevis* brains. Expression data analysis revealed *NeuroD1* gene activity in various brain regions, including olfactory bulbs, pallial regions of cerebral hemispheres, preoptic area, habenula, hypothalamus, cerebellum and medulla oblongata. In previous reports, *NeuroD1* gene expression has only been studied in embryonic and larval *Xenopus* stages (up to stage 48) in the context of primary neurogenesis and secondary neurogenesis, respectively [17], [23]–[26]. At larva stage, *NeuroD1* gene activity was identified in pallia, dorsal thalamus (habenula), pretectum, posterior tuberculum, nucleus of the medial longitudinal fascicle, mesencephalic optic tectum, torus semicircularis, tegmentum and medulla oblongata [23]. Thus, our study demonstrates that some of the previously identified larva brain regions maintained *NeuroD1* gene activity untill juvenile and adult stages. This is obvious for the pallial, habenula and cerebellar regions in which we detected the heaviest *NeuroD1* expressions. Importantly, our study also revealed that two additional post-metamorphic brain regions, compared to larva stage, expressed *NeuroD1*, namely the preoptic area and the hypothalamus. Previously, our laboratory performed studies to identify proliferation in the brain of both juvenile and adult *Xenopus* [9]. Interestingly, the patterns of *NeuroD1* expression were consistent with the majority of the proliferation zones that we mapped. In mammals, where most of NeuroD1 studies were performed, it was shown that structures such as the olfactory bulbs, cerebellum and hippocampus, maintained significant levels of *NeuroD1* mRNA expression at post-natal stages and also throughout adulthood in humans and mice [14], [27]–[30]. Recent studies in adult zebrafish telencephalon have also identified *NeuroD1* gene activity in the pallial region [31]. Wether or not the *Xenopus* NeuroD1 protein is regionally expressed at a similar level than the *NeuroD1* transcripts remains to be demonstrated. Unfortunately, due to the lack of available valid NeuroD1 antibodies, this could not be investigated.

In juvenile and adult *Xenopus* brains, we identified a large amount of *NeuroD1* expressing cells in the cerebellum, in particular in the granular layer. Interestingly, this strong *NeuroD1* gene activity is both conserved during *Xenopus* cerebellar development and across species. Indeed, in *Xenopus* and zebrafish larvae, a very strong *NeuroD1* expression in the developing cerebellum was previously detected [23], [32]. In mouse, at post-natal stages, *NeuroD1* was also clearly detected in both external and internal granular layers of the cerebellum, and the internal granular layers expression was shown to stably persist until adulthood [19], [27], [28]. In addition, systemic or conditional *NeuroD1* null mice experiments have shown that the absence of NeuroD1 leads to a lack of foliation and the complete loss of granular cells in the posterior half of the cerebellum, whereas a substantial number of granular cells survive and differentiate in the anterior lobules [19], [33], [34]. It will be interesting to investigate, in nonmammalian vertebrates, if there is a similar anterior-posterior differential requirement of NeuroD1 for granular cell maintenance.

The present study also shows that the medial pallium was another domain of abundant gene activity in juvenile and adult brains. In perfect agreement, we previously identified migrating cells and new born mature neurons in the medial pallium (D'Amico et al., 2011). Interestingly, the amphibian medial pallium is regarded as the homologue of the mammalian hippocampus [26], [35]. In other vertebrates, the hippocampus is also known to be one of the very few brain regions in which adult neurogenesis continues into adult stages of development, as demonstrated in reptiles, birds, fish and mammals [10], [36]–[43]. In mammals, after birth, *NeuroD1* is prominently expressed in the hippocampus, particularly in the granule cells of the dentate gyrus and pyramidal cells in CA1 and CA3 [27], [44]–[46]. As expected from the high expression level in the granule cell of the dentate gyrus, mice lacking the *NeuroD1* gene revealed striking abnormalities in the hippocampal formation [19], [20], [47]. Although the pyramidal layers appear normal, the mutant brains lack the dentate granule cell layer and have no organized dentate hilus [20]. More recently, it was found that overexpression of *NeuroD1* was sufficient to promote neuronal differentiation in adult hippocampal neural progenitors [48], whereas inducible *NeuroD1* gene ablation resulted in decreased survival and maturation of newborn neurons [29]. In our study, the presence of a high *NeuroD1* gene activity in the hippocampus of *Xenopus laevis* strongly suggest that NeuroD1 might also promote neuronal differentiation in this brain area in juvenile and adult animals.

In the course of this study, we identified in frog post-metamorphic brains new *NeuroD1* expression domains in the habenular, preoptic area and hypothalamic areas. To our knowledge, these *NeuroD1* expression domains have not been described in any adult vertebrate brain examined thus far. Wether or not the NeuroD1 factor has a critical function in these brain areas remains to be investigated. In particular, in preoptic and hypothalamic areas, it would be interesting to examine if the proliferative and neurogenic activities that we and others previously observed [9] are correlated, at cellular level, with up-regulation of a *NeuroD1* gene activity.

In post-metamorphic *Xenopus* brains, several arguments strongly suggest that *NeuroD1* expressing cells were not in a proliferation state. 1) In our detailed in situ hybridization analysis in juvenile and adult brains, we could never find *NeuroD1* expressing cells in the ventricular wall, in particular no co-localization with the radial glia or neural progenitor cells markers could be identified; 2) *NeuroD1* in situ hybridization combined with PCNA immunodetection did not allow identifying any co-localization of both factors; 3) Positive *NeuroD1*/BrdU double stainings were only found after long (14 days) post-BrdU administration survival time but never with short survival time (2 days). Therefore, we conclude that *NeuroD1* expressing cells in frog post-metamorphic brains are postmitotic. Interestingly, this feature seems to be conserved during *Xenopus* central nervous development. In *Xenopus* embryo, during primary neurogenesis, it was clear established that *NeuroD1* is expressed transiently in a subset of neurons in the central and peripheral nervous systems at the time of their terminal differentiation into mature neurons [17]. During *Xenopus* secondary neurogenesis, i.e. at early larval stages, *NeuroD1* gene expression was also excluded to the most ventricularly located cells in proliferation zones, in particular mitotic cells expressing *Ngnr-1* and *Delta1* genes [23]. Interestingly, in post-embryonic and adult zebrafish brains, *NeuroD1*-expressing cells were also identified from one to several cell rows away from the ventricular surface [31], [32], [49]. Taken together, *NeuroD1* expression studies in non-mammalian vertebrates have indicated that *NeuroD1*-expressing cells were post-mitotic at embryonic, larval and adult brain stages. Surprisingly, in murine *NeuroD1* expression was detected not only in post-mitotic but also in mitotic cells, as was evident in its expression in external granular layer of cerebellum and granule cells of the dentate gyrus during post-natal development. This observation suggested that NeuroD1 protein may have a unique role in proliferation and/or differentiation of granule cells of the cerebellum and dentate gyrus [19], [20], [27], [29], [30]. As *NeuroD1* expression was restricted to post-mitotic cells in the sub-ventricular zone and parenchyma, the present study suggests that NeuroD1 may play an important role in neuronal cell differentiation in the late stages of neurogenesis rather than proliferation stages in post-metamorphic *Xenopus* brain.

In an effort to understand the molecular cascade involved in frog adult neurogenesis, further investigations with others proneural and/or neurogenic factors have to be conducted in the future. During primary neurogenesis, and retinal neurogenesis, expression of *NeuroD1* is known to follow the expression of the bHLH gene *neurogenin-related-1* (*X-ngnr-1*), a vertebrate neuronal determination gene, also known as *Neurogenin 2* (*Ngn2*) in mammalians [17], [50], [51]. During primary neurogenesis, overexpression of *X-ngnr-1* induces formation of ectopic neurons in nonneuronal ectoderm and induces ectopic expression of *NeuroD1* ([50]. These data demonstrate that X-ngnr-1 and NeuroD 1 function to regulate successive stages of neuronal differentiation in the developing neural plate. Preliminary studies in our laboratory suggest that X-Ngnr-1 might not be a key factor during adult neurogenesis as we were not able to detect *X-Ngnr-1* expression, using stringent hybridization conditions, in any juvenile or adult brain areas, including regions with high proliferative and neurogenic capacities (data not shown). Wether or not other members of the *Neurogenin* family, such as *Xenopus Neurogenin 1* or *Neurogenin 3* [52], are able to compensate *X-Ngnr-1/Ngn2* expression in the adult neurogenetic network remains an open question.

## Materials and Methods

### Animals

For the present study, juvenile and adult *Xenopus laevis* of both sexes were used. Juveniles (NF stage 66) stage were classified according to Nieuwkoop and Faber (Nieuwkoop and Faber, 1967). All procedures involving animals were conducted in accordance with the guidelines of Ethical Committee at our institutions (University of Rennes 1, CNRS and INSERM) and in accordance with European Union regulations concerning the protection of experimental animals (Directive 86/609/EEC). The protocols were approved by the Ethical Committee CREEA (Comité Rennais d'Ethique en matière d'Expérimentation Animale) and performed under the supervision of authorized investigators (Permit number: 75-390). All steps have been taken to reduce suffering of animals. Animals were deeply anesthetized with 0.05% tricaine methane sulphonate (MS-222; Sigma) and killed by decapitation. The whole heads of juvenile frogs were fixed 2 hours in freshly prepared 0.5M, pH 7.4 phosphate–buffered saline (PBS) containing 4% paraformaldehyde (three specimens per stage). After two washes in PBS, brains were then carefully removed from the skull, post-fixed in fresh fixative overnight at 4°C and then stored, for no more than one week, in PBS untill sectioning.

### BrdU incorporation

To follow the fate of proliferating cells, juvenile *X. laevis* were anesthetized (as decribed above) and injected intraperitoneally with approximately 50 µL/g body weight of labeling reagent (Amersham Cell Proliferation kit; RPN20). After survival periods ranging between 2 days and 14 days post-injections, frogs were deeply anesthetized and killed as described above.

### In situ hybridization and immunohistochemistry

Brains were embedded in paraffin and sectionned coronally. Consecutive/adjacent thin sections of 8 µM thickness were placed on different slide sets allowing each individual brain to undergo in situ hybridization with different probes and/or antibodies (as described in the following). Sections were subjected to stringent in situ hybridization (ISH) as described [53]. The following ISH probes were used: *NeuroD1* [17], *Vimentin* [21] and *Sox2* [54]. For double ISH/immunodetections, the brains were first processed for ISH, then for immunocytochemistry. PCNA and BrdU immunodetections were performed as previously described [9]. All sections were photographed and analyzed under a Olympus PROVIS AX70 microscope with a digital camera (Olympus SP71), and a Nikon multizoom AZ100 macroscope with a DS-Ri1 color camera. Cell counting was doned manually under the microscope by two of the authors and by an observer unfamiliar with experiments. Red blood cells were clearly identified using 20× and 40× microscope objectives and were not counted.
